# Identification of the metabolites of isochlorogenic acid A in rats by UHPLC-Q-Exactive Orbitrap MS

**DOI:** 10.1080/13880209.2020.1822421

**Published:** 2020-09-23

**Authors:** Kaiyan Gong, Yuan Yang, Kailin Li, Lian Zhu, Xinjun Zhi, Wei Cai

**Affiliations:** a Hunan Provincial Key Laboratory of Dong Medicine, School of Pharmaceutical Sciences, Hunan University of Medicine, Huaihua, China; b Key Laboratory of Environmental Pollution Monitoring and Disease Control, Ministry of Education, School of Public Health, Guizhou Medical University, Guiyang, China; c Hunan Province Key Laboratory for Antibody-Based Drug and Intelligent Delivery System, Hunan University of Medicine, Huaihua, China

**Keywords:** LC-MS, 35-DiCQA, metabolomic, distribution, metabolic pathways, β-glucuronidase, relative abundance

## Abstract

**Content:**

Isochlorogenic acid A, one of the main components of *Duhaldea nervosa* (Wallich ex Candolle) A. Anderberg (Asteraceae), is a folk medicine used to treat a variety of diseases including fracture and rheumatoid arthritis. Despite its widespread use, the metabolism of isochlorogenic acid A *in vivo* has not been fully studied.

**Objective:**

An analytical strategy based on UHPLC-Q-Exactive Orbitrap MS is proposed for the detection and identification of the metabolites of isochlorogenic acid A in rats.

**Materials and methods:**

Six male Sprague-Dawley rats (180 ± 20 g) were randomly divided into two groups. Then, blood and tissue samples were obtained after oral administration of isochlorogenic acid A (200 mg/kg). All the samples were pre-treated by the Solid Phase Extraction (SPE) method. Next, the samples were analysed by UHPLC-Q-Exactive Orbitrap MS. Finally, the metabolites were identified based on the metabolomic workflow template.

**Results:**

A total of 33 metabolites were identified in rat plasma, with 30 of them being reported for the first time. The distribution of all metabolites in tissues was first investigated, three of them were widely distributed in liver, lungs, and kidneys. The corresponding reactions including methylation, hydrolysis, sulphate conjugation, glucuronide conjugation, as well as their composite reactions, are reported in this study.

**Discussion and conclusions:**

This method has wide-scale application prospects in the identification of metabolites. Considering that limited research has been conducted in this area, this study proposes metabolic pathways to further understand mechanisms of isochlorogenic acid A and the forms that are truly effective *in vivo*.

## Introduction

Isochlorogenic acid A (3,5-DiCQA) is a major component of *Duhaldea nervosa* (Wallich ex Candolle) A. Anderberg (Asteraceae) (Guan et al. 2017; Liu et al. 2018; Cai et al. 2020), which has served as a folk medicine in dispelling wind-chill, alleviating pain, promoting good circulation of the meridian, being collateral for anti-inflammatory, accelerating the union of fractures and treating a variety of conditions and diseases including fracture and rheumatoid arthritis (RA) (Xiao 1997, 2004; Long 2004). Previous studies have shown that 3,5-DiCQA has a wide range of physiological activities, such as cardiovascular protection, antioxidant, anti-inflammatory, and osteoblast proliferation, which might have a therapeutic effect in the treatment of fractures and RA (Naveed et al. 2018; Wang and Xiao 2019). With increasing attention on 3,5-DiCQA, it is very important to study its absorption, distribution, metabolism, excretion, and toxicity (ADMET); therefore, the identification of metabolites of 3,5-DiCQA can help in understanding its fate *in vivo* (Martínez-Archundia et al. 2018; Ferreira and Andricopulo 2019). However, to the best of our knowledge, the metabolites of 3,5-DiCQA have not been fully investigated. For instance, upon oral administration of the drug, only 2, 14, and 12 metabolites were detected in rat plasma, faeces and urine, respectively (Wang et al. 2017). Therefore, it is of great importance to study the metabolism of 3,5-DiCQA, to understand its mechanism and truly effective forms.

UHPLC-Q-Exactive Orbitrap MS with its advantages, such as high efficiency, sensitivity, and selectivity, is a very powerful technique for the detection and identification of chemical components in plant extracts or biological samples. This is because UHPLC provides fast and efficient separation and Q-Exactive Orbitrap MS provides accurate mass measurement and abundant MS^n^ fragment information (Clifford et al. 2003; Qiao et al. 2016; Cai et al. 2017).

This study was designed to identify the metabolites of 3,5-DiCQA from *D. nervosa* in rats by UHPLC-Q-Exactive Orbitrap MS. A total of 33 metabolites of 3,5-DiCQA were detected and identified based on high-resolution quality data analysis, chromatographic retention time and literature reference. Thirty of these metabolites were identified for the first time in the plasma. According to our research, the metabolic pathway of 3,5-DiCQA was proposed for the first time.

## Materials and methods

### Materials and chemicals

The deionised water was purchased from Watsons Water (Hong Kong, China). Both acetonitrile (Macklin, China) and formic acid (Fisher Scientific, NJ, USA) were of LC-MS grade, while methanol of LC grade was provided by Fisher Scientific (NJ, USA). Additional analytical reagents were obtained from Aladdin Industrial Corporation.

The standard compounds were identified by NMR spectrum, and the purity of no less than 98% was determined by HPLC-UV. Isochlorogenic acid A (3,5-DiCQA, 3,5-dicaffeoylquinic acid, Y-068-170903), isochlorogenic acid B (3,4-DiCQA, 3,4-dicaffeoylquinic acid, Y-069-180105), isochlorogenic acid C (4,5-DiCQA, 4,5-dicaffeoylquinic acid, Y-070-170515), neochlorogenic acid (3-CQA, 3-caffeoylquinic acid, X-014-170309), cryptochlorogenic acid (4-CQA, 4-caffeoylquinic acid, Y-067-180425), and chlorogenic acid (5-CQA, 5-caffeoylquinic acid, L-007-171216) were provided by ChengDu ManSiTe Bio-Technology (ChengDu, China).

### Animal experiments

Six male Sprague-Dawley rats (180 ± 20 g) were purchased from Hunan SJA Laboratory Animal Company in China and housed at a constant temperature of 24 ± 2 °C for a week of acclimatisation. The rats were randomly divided into two groups after fasting for 12 h with free access to water: group A, drug group for plasma and tissue; and group B, blank group for blank plasma and tissue. In accordance with the national law on the use of experimental animals, the experiment was approved by the Medicine Ethics Review Committee for Animal Experiments at the Hunan University of Medicine.

3,5-DiCQA was dissolved in deionised water and orally administered at a dose of 200 mg/kg body weight to group A, followed by blood samples collected from the posterior orbital venous plexus at 0, 0.5, 1, 2, and 4 h. Each blood sample was then centrifuged at 4000 rpm for 15 min to obtain the plasma. The tissues from the heart, spleen, lung, kidneys, liver, and brain were immediately dissected and flushed with cold biological saline. All plasma and tissue samples were stored at −80 °C until further pre-treatment.

### Standards solution preparation and sample pre-treatment

The standard solution including isochlorogenic acid A, B, C, neochlorogenic acid, cryptochlorogenic acid, and chlorogenic acid was dissolved in methanol and stored at 4 °C before analysis.

All samples were pre-treated using the Solid Phase Extraction (SPE) method. Initially, the SPE column (WondaSep C18, 200 mg/3 mL) was activated and equilibrated by flushing with 3 mL methanol and 3 mL water containing 0.5% formic acid. Then, the sample including 0.1 mL plasma or 1 mL supernatant of tissue homogenate was loaded on the preactivated column, followed by eluting with 3 mL water and 3 mL methanol containing 0.5% formic acid successively. Afterward, the methanol eluate was dried with a stream of nitrogen at room temperature to obtain the residue, which was again re-dissolved in 100 μL 5% acetonitrile-water and centrifuged at 13,000 rpm at 4 °C for 30 min. Finally, 2 μL of supernatant was injected into the UHPLC-Q-Excative Orbitrap MS for analysis.

The tissue was homogenised in 1:5 g/mL biological saline using a homogeniser (TL2010S, DHS Life Science & Technology Co., Ltd., Beijing, China). The homogenates were then centrifuged at 4000 rpm for 15 min to obtain the supernatant, which was further pre-treated by the SPE method as mentioned above.

### Enzymatic hydrolysis by β-glucuronidase

A total of 25 μL β-glucuronidase (400 μg/mL, Cloud-Clone Corp, Wuhan, China) was added to 100 μL of plasma, followed by the addition of 75 μL Tris HCL (0.1 M, pH 6.8). Afterward, the sample was sealed and incubated at 37 °C for an hour. Finally, the sample was pre-treated according to the SPE method as described above.

### Instruments and conditions

UHPLC analysis was performed using the Ultimate 3000 system (Dionex, Sunnyvale, CA, USA) equipped with an online vacuum degasser, a quaternary pump, and an automatic sampler. The samples were separated by an HYPERSIL GOLD C18 column (100 × 2.1 mm, 1.9 μm). Water with 0.1% formic acid (A) and acetonitrile (B) was used as the mobile phase. The flowing gradient at the rate of 0.3 mL/min was applied at 35 °C: 0–2 min, 2–5% B; 2–5 min, 5–8% B; 5–10 min, 8–15% B; 10–15 min, 15–18% B; 15–20 min, 18–40% B; 20–24 min, 40–80% B; 24–26 min, 80% B; 26–27 min, 80–2% B; and 27–30 min, 2% B.

ESI-MS^n^ analyses were performed on Q-Exactive Focus Orbitrap MS (Thermo Bremen, Germany) and the mass spectrometry was analysed using a negative heating electrospray ionisation source (Thermo Electron, Bremen, Germany). The optimised parameters were set as follows: the flow rate of sheath gas (nitrogen) was 30 arb and the flow rate of auxiliary gas was 10 arb; Spray voltage 3.0 kV; Capillary temperature 320 °C; and auxiliary gas heater temperature 350 °C; High- resolution MS scanning was conducted at full MS scan in the mass range of *m/z* 100–1200 with a resolution of 35,000 and MS^2^ at a resolution of 17,500 triggered by parallel reaction monitoring mode; nitrogen (99.999% purity) was used as a collision gas; and the energy was set to 30% normalised collision energy.

### Data processing and analysis

The Xcalibur version 4 workstation (Thermo Fisher Scientific, San Jose, CA, USA) was used to acquire all the raw data. The data was then analysed by Compound Discover version 3 with the metabolomics workflow template to detect the differential components between the drug and blank samples. The optimised parameters of metabolomics workflow template were set as follows: the minimum peak intensity was set at 10000; the maximum element count was set at C_30_ H_60_ O_20_ S_4_ N_10_ CL_4_; MS and MS^2^ with mass tolerances of 5 and 10 ppm, respectively; the tolerance of retention time was 12 s; and a different analysis was selected for post-processing.

## Results and discussion

### Analytical strategy

In order to profile and identify the metabolites of 3,5-DiCQA comprehensively, an analytical strategy was based on UHPLC-Q-Exactive Orbitrap MS. Initially, the comparable samples including plasma and tissue were prepared using the SPE method. The samples were then analysed by UHPLC-Q-Exactive Orbitrap MS acquired by full mass scanning mode to obtain the full mass raw data. The data was processed on the Compound Discover version 3 workstation using the metabolomics workflow template to detect the differential components between the drug and blank samples. Afterward, the sample was injected into the UHPLC-Q-Exactive Orbitrap MS again to obtain the fragmentation ions acquired by parallel reaction monitoring mode triggered by differential components detected above. Finally, the metabolites of 3,5-DiCQA were characterised based on accurate MS, fragmentation ions, retention time and bibliography.

### Identification of the metabolites of 3,5-DiCQA in rat plasma

**Figure 1.**
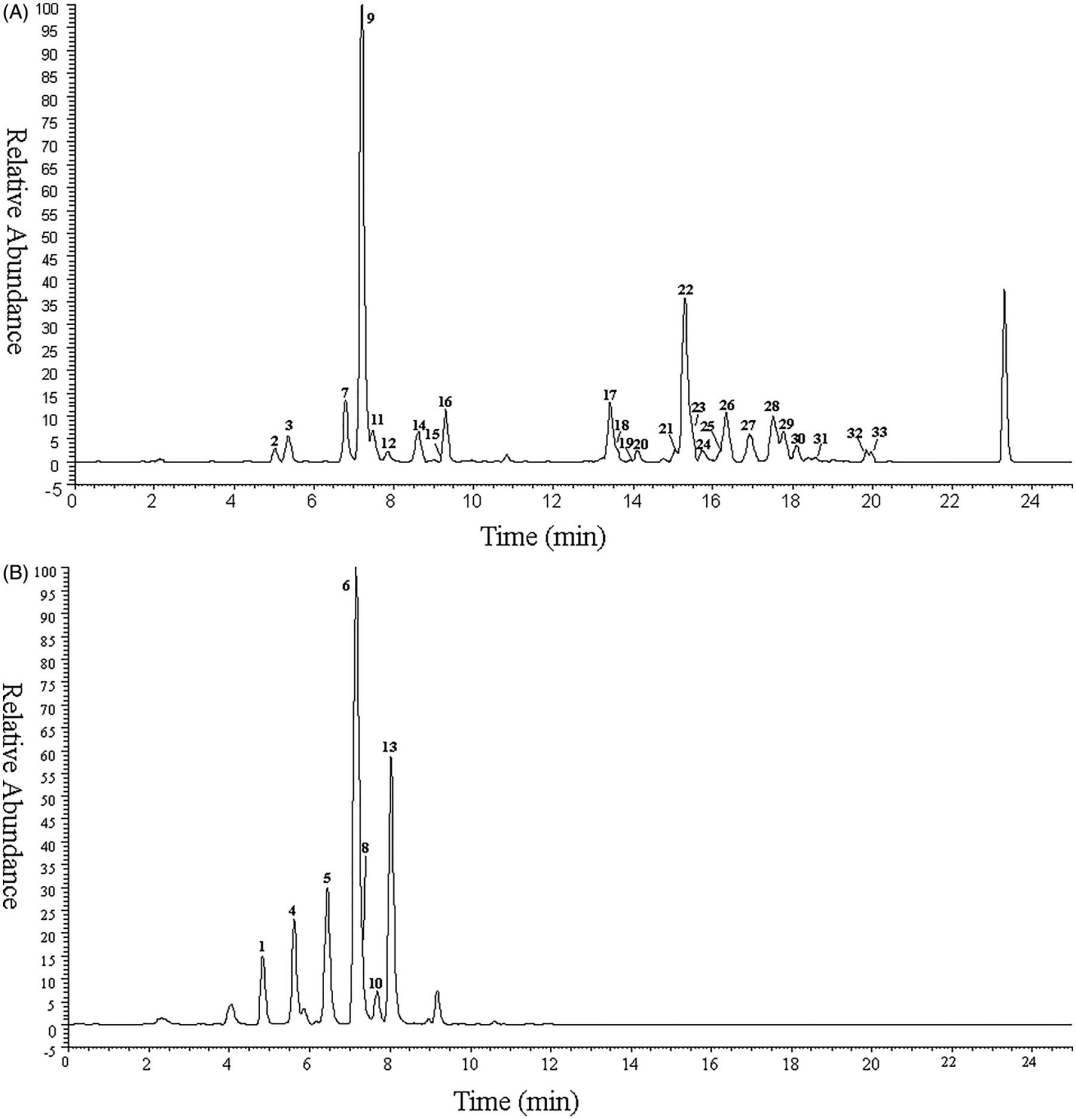
The high resolution extracted ion chromatography of isochlorogenic acid A metabolites (A) *m/z* 353.08780, 355.06706, 367.10345, 369.08272, 515.11950, 529.13515, 543.15079, 691.15159, 705.16724, 719.18289; (B) *m/z* 258.99180, 261.00745, 273.00744, 275.02310.

A total of 33 metabolites as well as the parent drug itself (3,5-DiCQA) were detected and identified based on UHPLC-Q-Exactive Orbitrap MS, while 30 of these metabolites were reported for the first time. The retention time and mass spectrometry data of these metabolites are shown in Table 1 and Supplementary Table S1, respectively. The high-resolution extraction ion chromatography of these compounds is illustrated in Figure 1.

**Table 1.** The retention time and mass spectrometric data of isochlorogenic acid A metabolites.

Peak	tR	Theoretical Mass *m/z*	Experimental Mass *m/z*	Error (ppm)	Formula [M-H]-	MS/MS fragment	Identification
1	4.83	261.00745	261.00720	−0.94	C_9_H_9_O_7_S	MS^2^[261]:137.0596 (100), 181.0497(52)	Sulphation of DHCA
2*	5.02	353.08780	353.08783	0.07	C_16_H_17_O_9_	MS^2^[353]:191.0547 (100), 179.0336 (76), 135.0436 (21)	3-CQA (neochlorogenic acid)
3	5.36	355.06706	355.06690	−0.31	C_15_H_15_O_10_	MS^2^[355]: 179.0337(100), 135.0439(25)	Glucuronide of CA
4	5.62	261.00745	261.00729	−0.60	C_9_H_9_O_7_S	MS^2^[261]: 181.0498(100), 137.0596 (27)	Sulphation of DHCA
5	6.44	258.99180	258.99161	−0.72	C_9_H_7_O_7_S	MS^2^[258]:179.0341(100), 135.0438(26)	Sulphation of CA
6	7.14	258.99180	258.99161	−0.72	C_9_H_7_O_7_S	MS^2^[258]:179.0340(100), 135.0439(18)	Sulphation of CA
7	7.19	355.06706	355.06700	−0.14	C_15_H_15_O_10_	MS^2^[355]:179.0341(100), 135.0438(36), 113.0229(26), 85.0281(16)	Glucuronide of CA
8	7.24	275.02310	275.02307	−0.10	C_10_H_11_O_7_S	MS^2^[275]:177.0548 (100), 195.0659(69)	Sulphation of DHFA
9*	7.47	353.08780	353.08765	−0.44	C_16_H_17_O_9_	MS^2^[353]:191.0555(100)	5-CQA (chlorogenic acid)
10	7.69	275.02310	275.02328	0.67	C_10_H_11_O_7_S	MS^2^[275]: 195.0659(100)	Sulphation of DHFA
11	7.75	367.10345	367.10236	−2.98	C_17_H_19_O_9_	MS^2^[367]: 193.0502(100)	3-FQA
12*	7.85	353.08780	353.08783	0.07	C_16_H_17_O_9_	MS^2^[353]:173.0441 (100), 179.0336 (82), 191.0547 (52), 135.0435 (30)	4-CQA (cryptochlorogenic aci)
13	8.02	273.00744	273.00739	−0.21	C_10_H_9_O_7_S	MS^2^[273]:193.0497 (100)	Sulphation of FA
14	8.55	355.06706	355.06700	−0.14	C_15_H_15_O_10_	MS^2^[355]: 179.0336(100), 135.0438(60), 113.0230(47)	Glucuronide of CA
15	9.21	353.08780	353.08936	4.40	C_16_H_17_O_9_	MS^2^[353]:191.0548 (100), 135.0435 (4), 179.0334 (3), 173.0078 (3)	Cis-5-CQA
16	9.27	369.08272	369.08221	−1.38	C_16_H_17_O_10_	MS^2^[369]: 193.0502(100), 113.0229(89), 85.0280(52)	Glucuronide of FA
17	13.41	691.15159	691.15210	1.54	C_31_H_31_O_18_	MS^2^[691]: 173.0444 (100), 179.0337 (52), 191.0547 (22)	Glucuronide of 3,4-DiCQA
18	13.59	691.15159	691.15063	−0.59	C_31_H_31_O_18_	MS^2^[691]:191.0553(100), 179.0335(61), 353.0883(51)	Glucuronide of 3,5-DiCQA
19	13.94	691.15159	691.14923	−3.41	C_31_H_31_O_18_	MS^2^[691]:191.0554(100), 179.0334(52)	Glucuronide of 1,5-DiCQA
20	14.08	691.15159	691.15143	0.57	C_31_H_31_O_18_	MS^2^[691]:173.0446(100), 179.0339(38), 353.0862 (32)	Glucuronide of 4,5-DiCQA
21*	15.07	515.11950	515.11987	0.72	C_25_H_23_O_12_	MS^2^[515]: 173.0441 (100), 179.0335 (89), 191.0547 (34), 135.0435 (14), 353.0865 (14)	3,4-DiCQA (isochlorogenic acid B)
22*	15.29	515.11950	515.11914	−0.70	C_25_H_23_O_12_	MS^2^[515]:191.0554(100), 179.0341(71), 135.0438(15), 353.0880(13)	3,5-DiCQA (isochlorogenic acid A)
23	15.49	719.18289	719.18237	−0.72	C_33_H_35_O_18_	MS^2^[719]:193.0501(100)	Dimethylation and glucuronide of DiCQA
24	15.72	515.11950	515.11926	−0.47	C_25_H_23_O_12_	MS^2^[515]:191.0546 (100), 179.0335 (72), 353.0865 (18), 173.0445 (11), 135.0435 (11)	1,5-DiCQA
25	16.17	719.18289	719.18311	0.31	C_33_H_35_O_18_	MS^2^[719]:193.0500(100)	Dimethylation and glucuronide of DiCQA
26	16.35	705.16724	705.16815	1.29	C_32_H_33_O_18_	MS^2^[705]: 193.0497(100), 161.0235(43)	Methylation and glucuronide of DiCQA
27	16.93	705.16724	705.16815	2.07	C_32_H_33_O_18_	MS^2^[705]:193.0495(100), 161.0228(57), 191.0554(53), 337.0538(44), 179.0335(32), 353.8655(26)	Methylation and glucuronide of DiCQA
28*	17.50	515.11950	515.11938	−0.23	C_25_H_23_O_12_	MS^2^[515]:173.0447(100), 179.0338(65), 191.0556(23), 353.0865 (22), 135.0439 (8)	4,5-DiCQA (isochlorogenic acid C)
29	17.78	719.18289	719.18262	−0.37	C_33_H_35_O_18_	MS^2^[719]:193.0499(100)	Dimethylation and glucuronide of DiCQA
30	18.10	719.18289	719.18378	1.24	C_33_H_35_O_18_	MS^2^[719]:193.0499(100)	Dimethylation and glucuronide of DiCQA
31	18.55	529.13515	529.13397	−2.23	C_26_H_25_O_12_	MS^2^[529]:191.0554(100)	3C, 5FQA
32	19.83	543.15079	543.14966	−2.10	C_27_H_27_O_12_	MS^2^[543]: 193.0499(100)	Dimethylation of DiCQA
33	19.98	543.15080	543.15186	1.95	C_27_H_27_O_12_	MS^2^[543]: 193.0499(100)	Dimethylation of DiCQA

*Confirmed with standard compounds.

Peaks 2, 9, 12, 21, 22, and 28 were accurately identified as 3-CQA, 5-CQA, 4-CQA, 3,4-DiCQA, 3,5-DiCQA, and 4,5-DiCQA, respectively by comparing the retention time, accurate MS, and fragmentation ions.

Peaks 15 and 24 showed the same pseudo-molecular ion [M–H]^−^ at *m/z* 353.08936 (4.40 ppm, C_16_H_17_O_9_), and 515.11938 (−0.23 ppm, C_25_H_23_O_12_), respectively as peaks 2, 9, 12, and 21, 22, 28. By comparing the fragment ion at *m/z* 191.0547 and the retention time as reported in another study (Liu et al. 2018; Cai et al. 2020), peaks 15 and 24 were preliminarily inferred as Cis-5-CQA and 1,5-DiCQA, respectively.

Peaks 11 and 31 were detected at 7.75 and 18.55 min, respectively with the deprotonated molecular ion at *m/z* 367.10236 (−2.98 ppm, C_17_H_19_O_9_), and 529.13397 (−2.23 ppm, C_26_H_25_O_12_), 14 Da (CH_2_) more than CQA (peaks 2, 9, 12) and DiCQA (peaks 21, 22, 28), suggesting that they were methylation products of CQA and DiCQA, respectively. Peaks 11 and 31 yielded the fragmentation ion at *m/z* 193.0502 and 191.0554, respectively; therefore, peaks 11 and 31 were tentatively identified as 3-FQA, and 3 C, 5FQA, respectively (Clifford et al. 2005; Jaiswal et al. 2014).

Peaks 32 and 33 eluted at 19.83 and 19.98 min generated the same deprotonated molecular ion [M–H]^−^ at *m/z* 543.150 (C_27_H_27_O_12_), 28 Da (2CH_2_) more than DiCQA, suggesting that they were dimethylation products of DiCQA (Yang et al. 2005), which were confirmed by the base peak ion at *m/z* 193.0499 in MS^2^ spectrum.

Peaks 5 and 6 were eluted at 6.44 and 7.14 min, respectively. Both displayed the same pseudomolecular ions [M–H]^−^ at *m/z* 258.99161 (−0.72 ppm, C_9_H_7_O_7_S), which yielded the base peak ion at *m/z* 179.034 by the loss of the SO_3_ moiety (80 Da). Their MS^2^ spectrum is consistent with the spectrum of caffeic acid (CA), therefore, they were tentatively characterised as sulphation products of CA. In the same way, peaks 1 and 4 were tentatively confirmed to be sulphation products of dihydrocaffeic acid (DHCA); peaks 8 and 10 were sulphation products of dihydroferulic acid (DHFA); and peak 13 was sulphation products of ferulic acid (FA).

Peaks 3, 7, and 14 produced their [M-H]^−^ at *m/z* 355.0669 (−0.31 ppm, C_15_H_15_O_10_), 355.0670 (−0.14 ppm, C_15_H_15_O_10_) and 355.0670 (−0.14 ppm, C_15_H_15_O_10_), respectively, and yielded the base peak ion at *m/z* 179.034 by the loss of 176 Da glucuronide. This data suggested that these metabolites were glucuronide conjugation. In the MS^2^ spectrum, ions at *m/z* 179.034 and 135.044 were observed, indicating that it could be attributed to CA. Therefore, they were tentatively inferred as glucuronide conjugation of CA. Peak 16 was eluted at 9.27 min with the pseudomolecular ions [M–H]^−^ at *m/z* 369.08221 (−1.38 ppm, C_16_H_17_O_10_), 14 Da (CH_2_) more than peaks 3, 7, and 14, suggesting that it might be the methylation and glucuronide conjugation of CA. This finding was confirmed by the natural loss at *m/z* 176.033 (C_6_H_8_O_6_, 369.08221→193.0502) in the MS^2^ spectrum. Therefore, peak 16 was tentatively identified as glucuronide conjugation of FA.

Peaks 17–20 were eluted at 13.41, 13.59, 13.94, and 14.08 min, respectively yielding the pseudomolecular ions [M–H]^−^ at *m/z* 691.1510 (1.52 ppm, C_31_H_31_O_18_) 691.15063 (−0.59 ppm, C_31_H_31_O_18_), 691.14923 (-3.41 ppm, C_31_H_31_O_18_) and 691.15143 (0.57 ppm, C_31_H_31_O_18_), 176 Da (C_6_H_8_O_6_) more than DiCQA, indicating that they might be glucuronide conjugation of DiCQA. Their fragmentation ions were similar to those of peaks 21, 22, 24, and 28, respectively; therefore, they were tentatively inferred as glucuronide conjugation of 3, 4-DiCQA, 3, 5-DiCQA, 1, 5-DiCQA, and 4, 5-DiCQA, respectively. In addition, peaks 26 and 27 were tentatively characterised as methylation and glucuronide conjugation of DiCQA, respectively. Lastly, peaks 23, 25, 29, and 30 were plausibly identified as dimethylation and glucuronide conjugation of DiCQA.

### Confirmation of metabolites using β-glucuronidase

In this study, the glucuronide conjugation metabolites proposed by mass spectrometry were further confirmed using β-glucuronidase hydrolysis. The peak area of glucuronide conjugation metabolites including peaks 3, 7, 14, 16–20, 23, 25–27, 29, and 30 was decreased, while the area of peaks 2, 9, 12, 21, 22, and 28 was increased after glucuronidase treatment. These results were consistent with the assignation of glucuronide conjugation metabolites based on mass spectrometry.

### Relative abundances of the metabolites in rats plasma

**Figure 2.**
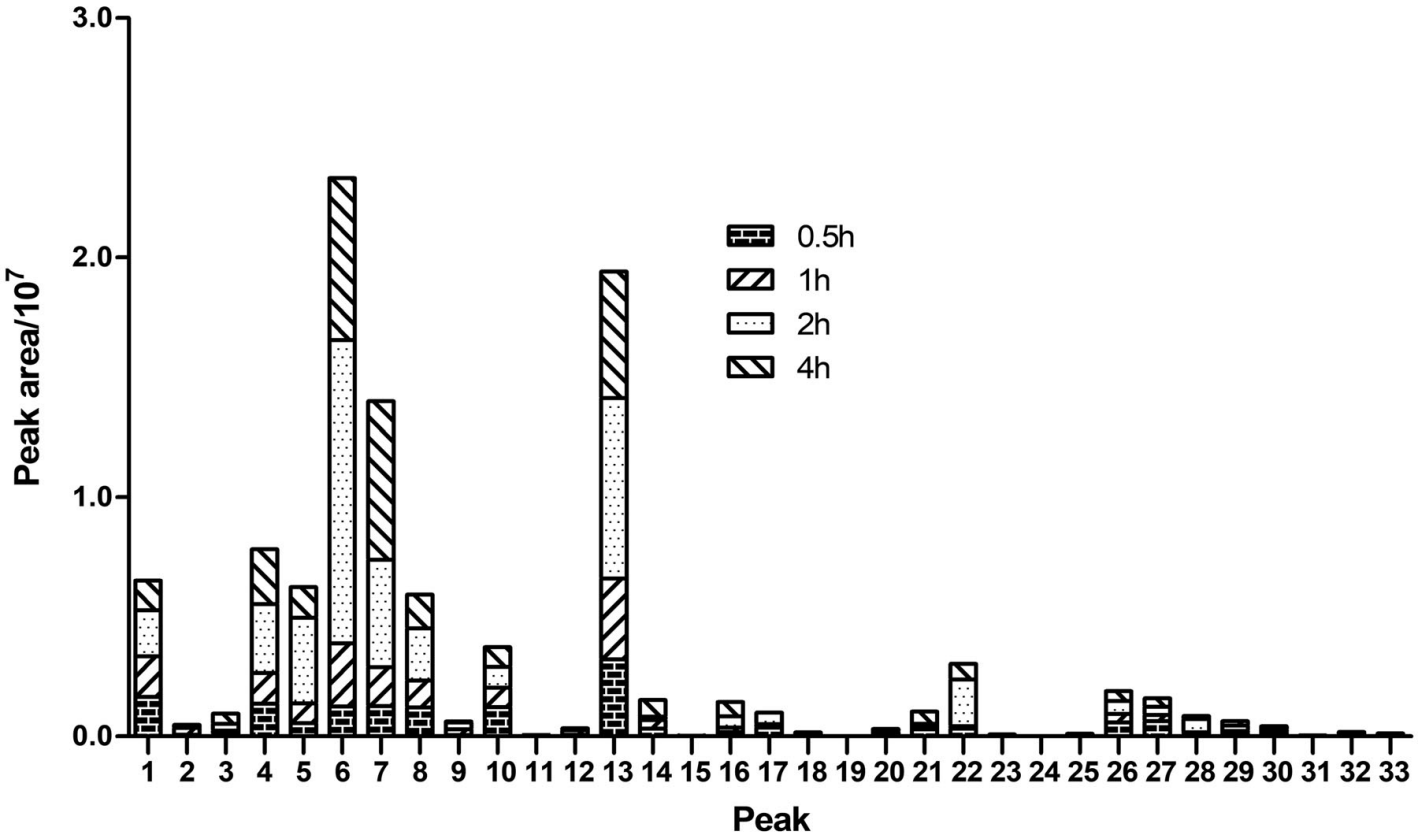
The relative abundance of metabolites in rat plasma.

The relative abundances of those metabolites of 3,5-DiCQA were compared by the peak area of those precursor ions in MS, which was displayed in Figure 2. In general, the metabolites of 3,5-DiCQA in 2 h plasma showed a relative abundance higher than those in 0.5, 1, an 4 h, while, the metabolites 6, 7, and 13 showed higher relative abundance than other metabolites in the same time point, which hinted that the composite reactions including hydrolysis + sulphation, and hydrolysis + glucuronide were the main metabolic reaction.

### Proposed metabolic pathways of 3,5-DiCQA

**Figure 3.**
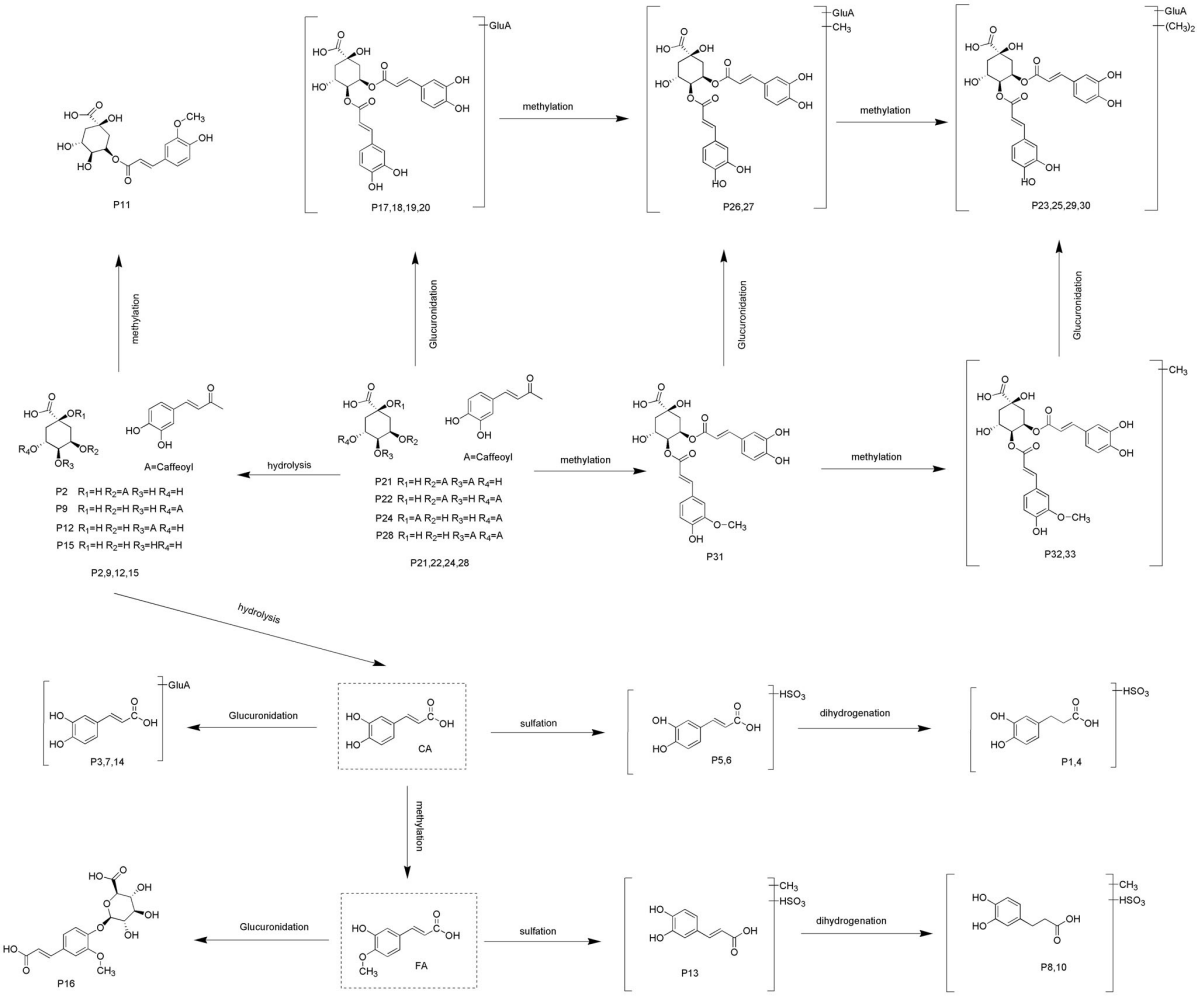
The proposed isochlorogenic acid A metabolic pathway in the rats plasma.

In this study, a total of 33 metabolites were unambiguously or tentatively identified based on UHPLC-Q-Exactive Orbitrap MS along with metabolomics and parallel reaction monitoring mode. The major metabolic pathways of 3,5-DiCQA in rat plasma are proposed in Figure 3. In general, 3,5-DiCQA undergoes three kinds of metabolic pathways. The first pathway involves the parent drug or its isomer including methylation (Peak 31), glucuronide conjugation (Peak 17–20), methylation with glucuronide conjugation (Peak 26 and 27), and dimethylation with glucuronide conjugation (Peak 23, 25, 29, and 30). The second pathway initially involves hydrolysis to gain CQA (Peak 2, 9, 12, and 15), followed by methylation (Peak 11). The third and the last pathway involves complete hydrolysis to yield CA, which is then immediately followed by a series of metabolic reactions including glucuronide conjugation (Peak 3, 7, and 14), sulphate conjugation (Peak 5 and 6), hydrogenation with sulphate conjugation (Peak 1 and 4), etc.

### Distribution of 3,5-DiCQA the metabolites in rat tissues

The distribution of all the metabolites in rats tissues was studied for the first time (shown in Supplementary Table S1). These results showed that a total of 4 metabolites which include peaks 7, 22, 32, and 33, were found in lung and liver; two metabolites that include peaks 32 and 33 were observed in the spleen; peak 22 was found in the heart; no metabolites were detected in the brain; 11 metabolites including peaks 7, 11, 14, 16, 22, 23, 25, 29, 30, 32, and 33 were detected in the kidney, indicating that the kidney might be the main metabolic organ. Peaks 22, 32, and 33 were distributed more widely than the other metabolites, suggesting that these metabolites might be the truly effective forms for exerting pharmacological effects.

## Conclusions

An analytical strategy based on UHPLCQ-Exactive Orbitrap MS technology with metabolomics and parallel reaction monitoring mode was established to detect and identify the metabolites of 3,5-DiCQA. A total of 33 metabolites were characterised in rat plasma, while 30 of these were reported for the first time. The corresponding reactions including methylation, hydrolysis, sulphate conjugation, glucuronide conjugation, and their composite reactions were all observed in this study. The results demonstrated that this practical strategy has the potential to be widely applicable for the detection and identification of metabolites. A total of 11 metabolites were detected and identified in organs. Among these, it is suspected that the parent drug and dimethylation products of DiCQA play an important pharmacological role *in vivo*. Therefore, further research is warranted to confirm these findings. In conclusion, this study proposes the metabolic pathways of 3,5-DiCQA for the first time, which will be helpful to further understand its mechanism and truly effective forms *in vivo*.

## Supplementary Material

Supplementary_Table_S1.docx
