# Supplementary material for: Identification of the metabolites of isochlorogenic acid A in rats by UHPLC-Q-Exactive Orbitrap MS
Source: Pharm Biol. 2020 Sep 23;58(1):992–8. doi: 10.1080/13880209.2020.1822421 (PMC7534263; doi:10.1080/13880209.2020.1822421)
Supplement: Supplementary_Table_S1.docx [file IPHB_A_1822421_SM8938.docx]

**Table 1S.** the present information of isochlorogenic acid A metabolites in rats

| Peak | t_R_ | Formula [M-H] | Identification | Heart | Liver | Spleen | Lung | Brain | Kidney | 0.5 h | 1 h | 2 h | 4 h |
| --- | --- | --- | --- | --- | --- | --- | --- | --- | --- | --- | --- | --- | --- |
| 1 | 4.83 | C_9_H_9_O_7_S | Sulfation of DHCA | - | - | **-** | - | - | - | + | + | + | + |
| 2 | 5.02 | C_16_H_17_O_9_ | 3-CQA (neochlorogenic acid) | - | + | - | + | - | + | + | + | + | - |
| 3 | 5.36 | C_15_H_15_O_10_ | Glucuronide of CA | - | - | - | - | - | - | + | + | + | + |
| 4 | 5.62 | C_9_H_9_O_7_S | Sulfation of DHCA | - | - | - | - | - | - | + | + | + | + |
| 5 | 6.44 | C_9_H_7_O_7_S | Sulfation of CA | - | - | **-** | - | - | - | + | + | + | + |
| 6 | 7.14 | C_9_H_7_O_7_S | Sulfation of CA | - | - | **-** | - | - | - | + | + | + | + |
| 7 | 7.19 | C_15_H_15_O_10_ | Glucuronide of CA | - | + | - | + | - | + | + | + | + | + |
| 8 | 7.24 | C_10_H_11_O_7_S | Sulfation of DHFA | - | - | - | - | - | - | + | + | + | + |
| 9 | 7.47 | C_16_H_17_O_9_ | 5-CQA (chlorogenic acid) | - | + | - | + | - | + | + | + | + | + |
| 10 | 7.69 | C_10_H_11_O_7_S | Sulfation of DHFA | - | - | - | - | - | - | + | + | + | + |
| 11 | 7.75 | C_17_H_19_O_9_ | 3-FQA | - | - | - | - | - | + | + | + | + | - |
| 12 | 7.85 | C_16_H_17_O_9_ | 4-CQA (cryptochlorogenic aci) | - | + | - | + | - | + | + | + | + | - |
| 13 | 8.02 | C_10_H_9_O_7_S | Sulfation of FA | - | - | - | - | - | - | + | + | + | + |
| 14 | 8.55 | C_15_H_15_O_10_ | Glucuronide of CA | - | - | - | - | - | + | + | + | + | + |
| 15 | 9.21 | C_16_H_17_O_9_ | Cis-5-CQA | - | - | - | - | - | - | + | + | + | - |
| 16 | 9.27 | C_16_H_17_O_10_ | Glucuronide of FA | - | - | - | - | - | + | + | + | + | + |
| 17 | 13.41 | C_31_H_31_O_18_ | Glucuronide of 3,4-DiCQA | - | - | - | - | - | - | + | + | + | - |
| 18 | 13.59 | C_31_H_31_O_18_ | Glucuronide of 3,5-DiCQA | - | - | - | - | - | - | - | - | + | - |
| 19 | 13.94 | C_31_H_31_O_18_ | Glucuronide of 1,5-DiCQA | - | - | - | - | - | - | - | - | + | - |
| 20 | 14.08 | C_31_H_31_O_18_ | Glucuronide of 4,5-DiCQA | - | - | - | - | - | - | + | + | + | - |
| 21 | 15.07 | C_25_H_23_O_12_ | 3,4-DiCQA (isochlorogenic acid B) | - | - | - | - | - | - | + | + | + | + |
| 22 | 15.29 | C_25_H_23_O_12_ | 3,5-DiCQA (isochlorogenic acid A) | + | + | - | + | - | + | + | + | + | + |
| 23 | 15.49 | C_33_H_35_O_18_ | Dimethylation and glucuronide of DiCQA | - | - | - | - | - | + | + | - | + | - |
| 24 | 15.72 | C_25_H_23_O_12_ | 1,5-DiCQA | - | - | - | - | - | - | + | - | + | - |
| 25 | 16.17 | C_33_H_35_O_18_ | Dimethylation and glucuronide of DiCQA | - | - | - | - | - | + | + | + | + | - |
| 26 | 16.35 | C_32_H_33_O_18_ | Methylation and glucuronide of DiCQA | - | - | - | - | - | - | + | + | + | + |
| 27 | 16.93 | C_32_H_33_O_18_ | Methylation and glucuronide of DiCQA | - | - | - | - | - | - | + | + | + | + |
| 28 | 17.50 | C_25_H_23_O_12_ | 4,5-DiCQA (isochlorogenic acid C) | - | + | - | - | - | - | + | + | + | + |
| 29 | 17.78 | C_33_H_35_O_18_ | Dimethylation and glucuronide of DiCQA | - | - | - | - | - | + | + | + | + | + |
| 30 | 18.10 | C_33_H_35_O_18_ | Dimethylation and glucuronide of DiCQA | - | - | - | - | - | + | + | + | + | - |
| 31 | 18.55 | C_26_H_25_O_12_ | 3C, 5FQA | - | - | - | - | - | - | + | - | + | - |
| 32 | 19.83 | C_27_H_27_O_12_ | Dimethylation of DiCQA | - | + | + | + | - | + | + | + | + | - |
| 33 | 19.98 | C_27_H_27_O_12_ | Dimethylation of DiCQA | - | + | + | + | - | + | + | - | + | - |
